# Integrating primary care and social services for older adults with multimorbidity: a qualitative study

**DOI:** 10.3399/BJGP.2020.1100

**Published:** 2021-09-07

**Authors:** Hajira Dambha-Miller, Glenn Simpson, Lucy Hobson, Doyinsola Olaniyan, Sam Hodgson, Paul Roderick, Simon DS Fraser, Paul Little, Hazel Everitt, Miriam Santer

**Affiliations:** School of Primary Care, Population Sciences and Medical Education, University of Southampton, Southampton.; School of Primary Care, Population Sciences and Medical Education, University of Southampton, Southampton.; School of Primary Care, Population Sciences and Medical Education, University of Southampton, Southampton.; General Medicine Department, Hinchingbrooke Hospital, North West Anglia NHS Trust, Huntingdon.; School of Primary Care, Population Sciences and Medical Education, University of Southampton, Southampton.; School of Primary Care, Population Sciences and Medical Education, University of Southampton, Southampton.; School of Primary Care, Population Sciences and Medical Education, University of Southampton, Southampton.; School of Primary Care, Population Sciences and Medical Education, University of Southampton, Southampton.; School of Primary Care, Population Sciences and Medical Education, University of Southampton, Southampton.; School of Primary Care, Population Sciences and Medical Education, University of Southampton, Southampton.

**Keywords:** ageing, caregivers, multimorbidity, primary health care, social support, social work

## Abstract

**Background:**

Growing demand from an increasingly ageing population with multimorbidity has resulted in complex health and social care needs requiring more integrated services. Integrating primary care with social services could utilise resources more efficiently, and improve experiences for patients, their families, and carers. There is limited evidence on progress including key barriers to and drivers of integration to inform large-scale national change.

**Aim:**

To elicit stakeholder views on drivers and barriers of integrated primary care and social services, and highlight opportunities for successful implementation.

**Design and setting:**

A qualitative interview study.

**Method:**

Semi-structured interviews with maximum variation sampling to capture stakeholder views across services and professions.

**Results:**

Thirty-seven interviews were conducted across England with people including GPs, nurses, social care staff, commissioners, local government officials, voluntary and private sector workers, patients, and carers. Drivers of integration included groups of like-minded individuals supported by good leadership, expanded interface roles to bridge gaps between systems, and co-location of services. Barriers included structural and interdisciplinary tension between professions, organisational self-interest, and challenges in record sharing.

**Conclusion:**

Drivers and barriers to integration identified in other contexts are also present in primary care and social services. Benefits of integration are unlikely to be realised if these are not addressed in the design and execution of new initiatives. Efforts should go beyond local- and professional-level change to include wider systems- and policy-level initiatives. This will support a more systems-wide approach to integrated care reform, which is necessary to meet the complex and growing needs of an ageing multimorbid population.

## INTRODUCTION

The population is ageing and by 2035 the absolute number of people aged ≥65 years in England is projected to rise by over 48%.^1^ At least 54% of the UK population aged >65 years is currently living with ≥2 long-term health conditions (multimorbidity), and this will exceed 66% over the next decade.^2^ Multimorbidity is associated with reduced functional status, increased healthcare utilisation, longer hospital stays, and more complex psychosocial needs. The implications are substantial health and social support for personal care needs, and assistance with mobility, housing, or financial support alongside disease management. Consequently, demand for health and social services is likely to increase further, adding to the strain on existing services. To address this, the *NHS Long Term Plan* proposed more integrated health and social care services.^3^^,^^4^ Given that patients with long-term conditions account for approximately 50% of all GP appointments, and with multiple long-term conditions increasingly becoming the norm, effective integration with social services could potentially release capacity in primary care, reduce duplication, increase efficiency, and improve experiences for patients.^5^ Integration can contribute to better physical and mental health outcomes, and a recent review of 27 integrated care programmes for adults with chronic diseases reported increased treatment adherence and lower health costs.^6^ An umbrella review of 50 systematic reviews suggested that integrating health and social care can limit costs by reducing emergency admissions and readmissions, and enable care within patients’ homes for as long as possible.^7^

The structure and funding of social care markedly differs from primary and secondary care. In England, 152 local authorities manage social care, with statutory responsibilities for assessing individuals’ needs, commissioning services, and safeguarding.^8^ Over 90% of adult social services are provided by private and voluntary organisations.^9^ Unlike health care, social care is not a universally free service; rather, it is funded through a mixture of central government grants, council tax, business rates, and charging people who can afford to pay. These structural and funding differences present a complex challenge in the effort to integrate services.^10^ Furthermore, there is limited conceptual understanding of what closer alignment between these services might look like in practice, and integrated care models have been implemented with limited evidence of effectiveness. For example, ‘Vanguard’ sites were established in 2015 to test models of integrated care, followed by interventions such as ‘social prescribing’, ‘care navigation’, and the Integrated Care Systems programme; none of these underwent thorough evaluation before national rollout.^11^^,^^12^ There is a paucity of studies, especially those employing qualitative methods, that investigate progress towards integration specific to primary care and social services in England. This study explored the topic from the perspective of stakeholders to elicit responders’ views on the progress of, drivers of, and barriers to existing integration strategies. It highlighted examples of successful implementation, providing insights that could inform efforts to achieve closer integration.

**Table:** How this fits in

There is a paucity of research examining progress towards integration of primary care and social services in England. This study found that integration is progressing slowly and is unlikely to be fully realised if new initiatives are not designed and implemented holistically to include change beyond the local and professional levels. Future solutions should focus on the macro-scale at the higher organisational and strategic levels of health and social care to ensure a systems-wide approach to reform. The findings are relevant to GPs, by offering insights into factors that facilitate effective integration with social services. Integrated health and social care has the potential to release capacity in primary care.

## METHOD

### Design

While this research is approached from a primary care and social care perspective, it is evident that any study of integration must be framed within a systems-wide context,^13^ which takes account of all dimensions of health and social care. Using this holistic approach, a qualitative semi-structured interview study was conducted with key stakeholders delivering and using these services.

### Recruitment and sampling

Purposive sampling was employed to capture a range of participant views. Sixty-three individuals were sent an email invitation after expressing interest through adverts seen on social media, community centres, the dedicated project website, charity newsletters, and through word of mouth. Of these, 37 responded and were interviewed. Participants were recruited from primary care, adult social services, secondary care, third sector providers, the care home sector, public health, housing, health and wellbeing board, patients, and carers. Given the complex structure of health and social care, an iterative recruitment approach was necessary. A snowball sampling technique was used from the initial round of 10 interviews to identify further participants.

### Data collection

Telephone interviews were conducted between June and September 2020, each lasting between 30 and 60 min. Interviews were conducted by telephone instead of face-to-face owing to COVID-19 restrictions. An interview schedule was designed (Box 1) covering broad questions to enable similar subjects to be addressed across the sample. A flexible approach ensured related subjects of importance could be raised. Design of the interview schedule was informed by the study aim, an earlier scoping review, and team members’ expertise, and then tested before use. Later interviews did not identify significant additional codes, views, or experiences, so it was concluded that data saturation had been achieved. Interviews were audiorecorded, transcribed verbatim, and anonymised. None of the interview participants were known to the interviewers.

**Box 1.** Interview schedule

**Interviews with health and social care staff** Describe your role in delivering integrated or joined-up health and social care, and the links you have with other professionals and organisations What aspects of integrated or joined-up health and social care have worked well, and why? What aspects of integrated or joined-up health and social care worked less well, and why? What changes would you like to see in these services, in terms of making them more integrated or joined up? **Interviews with patients, relatives, and carers** Please tell me about the reasons why you began using health and social care services? What aspects of these services worked well together for you? What services did not work well? What changes would you like to see in these services, especially in terms of making these services more integrated or joined up?

### Data analysis

An iterative form of inductive thematic analysis was employed. The first stage involved becoming familiar with the data.^14^ Three team members independently coded a sample of transcripts from the first round of interviews, then met to discuss initial interpretations until consensus was reached, leading to the formulation of a coding framework. Subsequent rounds of coding were conducted with further iterative refinement of the framework. Recurring patterns in the data were identified, leading to the development of themes.

Throughout the analytical process, a form of constant comparative analysis was used to identify key differences or similarities in the data, between professions, sectors, or geographies, for example. Alternative perspectives that may have challenged the authors’ interpretations were also searched for. This process of deviant case analysis reduced the risk of bias and added rigour to the analytical conclusions. A summary of the findings was sent to a sample of four participants to ensure views had been adequately captured.

QSR NVivo software (version 12) was used to manage the data, and the Consolidated Criteria for Reporting Qualitative Research (COREQ) checklist guided reporting.

### Data availability

Data are available from authors with a reasonable request.

## RESULTS

Thirty-seven people were interviewed: 23 females and 14 males. Participants comprised seven patients/carers and 30 professionals, from across care sectors and regions of England (Box 2). The analysis identified three overarching themes and additional subthemes, which are discussed narratively below with supporting quotes.

**Box 2.** Participant characteristics

**ID**	**Sex**	**Role/Job title**	**Sector**	**County/City**
1.	F	Carer/relative	Member of the public	Berkshire
2.	F	Carer/relative	Member of the public	Berkshire
3.	F	Carer/relative	Member of the public	Oxfordshire
4.	F	Carer/relative	Member of the public	Northumberland
5.	M	Relative	Member of the public	Leicester
6.	F	Relative	Member of the public	London
7.	M	Patient	Member of the public	Oxfordshire
8.	M	Senior social worker	Local government, adult social care	Wiltshire
9.	F	Social worker	Local government, adult social care	Peterborough
10.	M	Social worker	Local government, adult social care	Cambridge
11.	F	Hospital integration manager	Local government, adult social care	Wiltshire
12.	F	Customer care manager	Local government, adult social care	Cambridgeshire
13.	F	Assistant team manager	Local government, Department of Community Services	Wiltshire
14.	M	Director of Public Health	Local government, Public Health	Hampshire
15.	M	Private letting accommodation manager	Local government, Housing Department	Oxfordshire
16.	M	Head of housing	Local government, Housing Department	Oxfordshire
17.	F	Elected member, former chair of health and wellbeing board	Local government	Northumberland
18.	F	Service lead	Primary care/community services	Somerset
19.	F	GP	Primary care	Somerset
20.	M	Care navigator	Primary care	Hampshire
21.	M	GP	Primary care	Dorset
22.	F	Interface GP and medical advisor for ambulatory care	Primary care	Oxfordshire
23.	F	Community nurse	Secondary care	Northumberland
24.	F	Integrated discharge service lead	Secondary care	Somerset
25.	F	Care coordinator	Secondary care	Northumberland
26.	M	Medical advisor Oxfordshire urgent care services	Secondary care	Oxfordshire
27.	F	Charity lead	Voluntary sector	Leicester
28.	F	Volunteer centre manager	Voluntary sector	Hampshire
29.	M	Chief executive	Voluntary sector	London
30.	M	Senior health influencing manager	Voluntary sector	London
31.	F	Registered home care manager	Voluntary sector	Northumberland
32.	M	Lay care home/PLACE assessor and volunteer	Voluntary/statutory sector	Hampshire
33.	F	Chair	Voluntary/statutory sector	Hampshire
34.	M	Volunteer/ambassador	Voluntary/statutory sector	Hampshire
35.	F	Care home manager	Care home sector	Lancashire
38.	F	Care home manager	Care home sector	Northumberland
37.	F	Head of quality	Care home sector	Yorkshire

### Theme 1: Facilitators of primary care and social services integration

Participants highlighted factors facilitating integrated care for older people experiencing multimorbidity as follows:

#### Individuals and teams driving integration

Participants identified the role of key individuals or teams as innovators and drivers of integration:

*‘There is a brilliant geriatrician … who had this proactive approach and worked very well also with her colleagues in GP practices … She was trying to coordinate things across the system, and … it really works well. A lot of it though is dependent on charismatic individuals.’*

(Participant [P]14, local government, Public Health)

Interviewees credited individuals driving integration with recognising the benefits of empowering others and creating a culture that encourages initiative, enabling frontline professionals to develop joined-up solutions:

*‘It’s just the people on the ground feeling they’ve got the trust, and the freedom and the expectation to come up with ideas when they’re seeing that things could work better … that really comes from Dr K empowering me and my team, and those around her.’*

(P18, female, primary care/community services)

Team building was identified as essential to integration, described by an interviewee as an incremental developmental process:

*‘We built things at a steady pace … it’s constant work … started with a small core district nurse GP social prescriber and our hub coordinator nurse, and we’ve built from there. So rather than waiting for the whole set to be ready, we’ve got started, we’ve built a good, strong core team. Then, social care were willing to come into that functional group … mental health have come in.’*

(P19, primary care)

Some participants stressed that integration requires leadership across all levels and sectors of health and social care, especially to ensure that resources align with demand:

*‘Having the willingness of the right people at the right level to say, “OK, so maybe the capacity is in the wrong place.”’*

(P24, secondary care)

#### Interface roles

Participants identified the importance of non-clinical and clinical coordination roles, with various titles such as care navigators or integrated care coordinators, who work at the intersections where primary care, secondary care, and social services meet:

*‘In the GP surgery, they had their own team who were involved more with social issues … and they called them health coordinators. It was one of the workers there helped me* [an adult social worker] *to organise sorting out his* [an older adult client] *house, because it was in a bit of a state.’*

(P8, local government, adult social care)

Operationally, these interface roles were viewed as critical in facilitating integration among service providers by bridging gaps across sectoral boundaries:

*‘Social prescribers are the linchpin of linking primary* [care] *with adult services … the enabler that gives that bit of confidence … the bridge between the two.’*

(P28, voluntary sector)

Care coordinators were described as crucial in addressing the everyday social care and psychosocial needs of older people experiencing multimorbidity, once discharged into community settings:

*‘Someone had gone home, a daughter had gone on holiday to Italy with the keys … The care navigator said, “With the say-so of the patient … can we get a new lock put on your door with a new set of keys, and we can discharge you home?” Actually, non-clinically, looking at the issue of saying: “OK, you’ve sorted out the clinical, let me sort out the social and community aspect.”’*

(P33, voluntary/statutory sector)

Care coordinators were perceived as system navigators by carers and patients, providing support and advice when navigating the complex systems of health and social care:

*‘There’s a real need for maybe an elderly care coordinator … within a hub of GP practices that you have somebody that’s responsible for the elderly people in your community … and maybe trying to ensure that they are in touch with the people that they need to be.’*

(P3, carer/relative)

Another carer said:

*‘when you’ve got four or five different things going on, you think … if there was just one person and we spoke to them and said, “Can this happen?” That would make a massive difference.’*

(P2, carer/relative)

Having in place a layer of professionals located at potential ‘pinch points’ in the systems of health and social care was identified as not only significant in terms of reducing delays and blockages, but also in enabling seamless care transition across sectoral boundaries:

*‘Some GPs are incredibly helpful, some aren’t. Some won’t share any information with us* [adult social care]. *Every surgery has got a clinical coordinator. If you’ve got a slightly risky discharge* [from an acute hospital], *we would phone them and say,* “Mrs Smith is coming home with a four times daily care package, can you just pop out and see them?” … If they’ve got any concerns about their clients who are admitted into hospital, they’re very quick to phone us and say, give us the back story.’

(P11, local government, adult social care)

#### Co-location and collaboration

Participants identified co-location as a spatial and social enabler of integration. This concept is understood as a shared working environment where professionals from various disciplines can interact and collaborate:

*‘*[To improve coordination of hospital discharge among partners, the integrated discharge service lead] *tried to get their input into how we could change things in their environments, as well as processes … we all stopped for an hour and we did all sorts of things. That was mainly just to try and get them to mix, talk to each other from different organisations … That was helpful, just so that they could then appreciate where each other were … and also for me, it then helps to see how each one works differently.’*

(P24, secondary care)

Shared working spaces were identified as facilitating interprofessional relationship building and bonds of trust, which are essential to establishing sustainable, integrated working arrangements:

*‘It is about those, literally, working in the same offices. I think it’s also about relationships … if somebody lands on somebody’s door, we’re now saying, “Actually, it might not be the right place for us, but actually we know who can support you and where you can go.”’*

(P18, primary care/community services)

The emergence of a shared multidisciplinary team ethos in co-located spaces appeared to create an environment that enabled professionals to challenge one another and engage in difficult conversations about appropriate options for patient care:

*‘It took probably six to twelve months, I would say, for us to …* [become a joined-up interprofessional team]. *What we do now, we go into meetings and we really challenge one another, but we do that from the point of view: “I’m not angry with you … I’m just doing my role.” It was really difficult at first … Now, I think there’s a level of respect there for each other.’*

(P11, local government, adult social care)

Translating a vision of integrated working into practice requires stakeholders to agree a plan of action of how they will collaborate:

*‘I went to visit* [a] *hospital down in Somerset … What they did actually … is they went and sat everybody in a basement for a week from everywhere, all of the organisations, and said, “We are not leaving until we’ve come up with a plan to work together.” From what I could tell … it has had a huge impact on them as a county.’*

(P24, secondary care)

### Theme 2: Where integration occurs

Participants highlighted the multilevel nature of health and social care integration. This study’s data suggest that efforts to drive forward integration are mainly focused on two levels. First, there are micro-level clinical initiatives that aim to join up care at the point of delivery to the patient:

*‘The acute trust were really keen on having social work presence at the front door … they ring us* [adult social care] *and we’ll be down there within … four hours, is the agreement.’*

(P11, local government, adult social care)

Another participant commented:

*‘* [the GP practice] *employs a discharge liaison person to work at … our local hospital … We’ve got that really nice link of somebody … who’s working in the hospital.’*

(P18, primary care/community services)

Second, integration takes place at the meso-level in the form of joint arrangements between organisations:

*‘We all* [adult social care] *work quite well with mental health because there’s jointly funded posts … it’s not just looking at things from one angle, it’s looking at it from, I guess, a more holistic point of view. What it means for the person in their life, rather than what it means for the person with their social care and what it means to them with their medical needs. It’s smoother.’*

(P12, local government, adult social care)

*‘I* [senior GP] *no longer have just GPs in the practice, but I have paramedics, pharmacists, and nurse practitioners, practice nurses … we’ve got a physio within the practice now — a social prescriber. I think these are major steps forward.’*

(P21, primary care)

Operationally, integration requires effective interprofessional collaboration across levels by bringing together different health and social care sectors:

*‘So, it’s a really good two-way thing. That unplanned admissions team is absolutely essential to the way we* [social prescribing community development service] *work, and our working together is really crucial. The MDT* [multidisciplinary team] *meetings that came alongside that … which was us, the unplanned admissions team, district nurses, our discharge liaison.’*

(P18, primary care/community services)

### Theme 3: Tensions

A number of tensions in progressing integration were identified. Structural tensions were an inherent feature of the complex multilayered configuration of health and social care:

*‘The system is not really well designed to support that integrated working. So, someone in the hospital … They’ll really concentrate as hard as they can on that period, but then once that’s finished, they move on to the next person. Even the language and even the funding structures that support that approach, to a lesser or greater degree,* [are] *depending on where you are.’*

(P30, voluntary sector)

Health and social care are delivered through a series of separate systems, which in itself is an inhibitor of integration:

*‘It’s about problem-solving rather than just retrenching to your own bit and lobbing stones. I think culturally, that’s been quite difficult because our systems are set up quite adversarially in a way … everybody’s got their … own little silo to protect.’*

(P17, local government)

These separate structures can lead to tensions emerging among organisations. Most frequently, participants identified the tendency of organisations adopting a silo mentality, which emphasises internal priorities over potential benefits arising from external collaboration:

*‘There is a huge amount of siloed thinking. The hospitals are very good at protecting their areas of expertise by using NICE* [National Institute for Health and Care Excellence] *guidance. This was about the hospital making sure they kept control of a particular speciality.’*

(P21, primary care)

Organisational self-interest and protectionism, which is an institutional response involving organisations protecting their interests and retaining control over specialisms, was identified as a further barrier to integration:

*‘I deal with* [a neighbouring hospital trust] *quite a bit … The systems there are much slicker because you don’t have this territorialism.’*

(P26, secondary care)

Poor communication inhibited integration, both between and within organisations:

*‘There needs to be better communication as well, between the GP surgery and between ourselves* [adult social care] *… when we have safeguarding concerns and there’s a … professionals meeting — sometimes they don’t turn up … and there’s constant arguments between us and the GP practice, and then it just becomes really draining.’*

(P9, local government, adult social care)

Another well-documented tension raised by participants was the inability to share records between service providers owing to factors such as systems incompatibility and uncertainty over legal requirements relating to information sharing:

*‘We have no integration between these different systems. I think this is everybody’s biggest bugbear. So much time would be saved by being able to dive into each other’s medical records and look at what’s going on.’*

(P26, secondary care)

For patients, carers, and families, navigating the series of systems that constitute health and social care provision can be a frustrating challenge:

*‘We felt that we were having to speak to so many different people. You’d go to one person and they’d deal with that bit, and the next person would deal with another bit, and another person.’*

(P2, carer/relative)

Participants highlighted how tensions among health and social care actors were playing out across spatial scales:

*‘So, the other doublespeak is that they want policies … to be developed from the bottom up, but universally it’s always top down because that’s where the funding decisions come from, and until we truly give the money to* [local primary care] *networks, for them to absolutely decide what their priorities are, it’s never really going to change.’*

(P21, primary care)

Some participants argued for more practice- and solutions-based approaches that are localised, and emerge at the clinical and professional levels from empowered individuals with the autonomy to act:

*‘Surely somebody in the top tables are trying to figure out how this can happen … sometimes it’s just the people on the ground feeling they’ve got the trust and the freedom and the expectation to come up with ideas when they’re seeing that things could work better.’*

(P18, primary care/community services)

Another participant said:

*‘We just have to ensure that the teams communicate well and that the teams have a feeling of autonomy. My worry is that this* [the integration agenda] *has been approached in a rather piecemeal fashion.’*

(P21, primary care)

## DISCUSSION

### Summary

This study explored stakeholder views on progress of, drivers of, and barriers to existing integrated care initiatives within primary care and social services. Like-minded individuals were often key drivers of integration supported by strong leadership. Interface roles were emphasised to bridge gaps between providers, facilitate seamless service provision, and support patients and carers to navigate the complex health and social care infrastructure. Co-located spaces acted as creative arenas, enabling formal and informal integration. In practice, integration was mainly focused on micro-level frontline clinical initiatives to facilitate interdisciplinary working among professionals, while concomitantly there were tensions in progressing towards greater systems-wide integration. These tensions were viewed as an inherent feature of health and social care delivery, which was a series of disparate organisational structures and systems where there was limited learning and progress from previously tested models.

### Strengths and limitations

One strength of this study is the large sample of participants from a diverse range of professions, regions, sectors, and scales of integration, including strategic-level management and frontline clinical and nonclinical service providers alongside service users and their carers. Semi-structured interviewing enabled open-ended probing and in-depth exploration of participants’ views, allowing for a more holistic understanding of integration.

However, the use of snowball sampling and the study’s reliance on professionals and laypersons voluntarily opting in to the research may have introduced an element of self-selection. COVID-19 limited methodological options, and, given the restrictions imposed on social interaction, interviews had to be conducted by telephone. This may have been a factor in the small sample of patients. It is plausible that different accounts may have been obtained with in-person interviews. Understandably, the responses of participants may to some degree have been framed by the impact of the ongoing pandemic on contemporary health and social care practice.

### Comparison with existing literature

These results are consistent with earlier work on integration, but few previous studies have examined primary care and social services.^13^^,^^15^ It is concerning that many of the factors identified as important in this study have been highlighted in previous literature in other fields of integrated care, yet remain poorly applied in newer models of integration. This may well be contributing to the inadequate pace and progress of integrated services. The pivotal role of leaders and leadership in driving and sustaining integrated working are highlighted in earlier studies but are still not sufficiently prioritised in current care models or policymaking.^16^^,^^17^ This study highlights the importance of innovative individuals and teams who, if empowered, can drive forward integration. The finding that co-location is effective in bringing together professionals from multidisciplinary backgrounds to deliver integrated care needs to be considered in light of the recent move towards remote collaboration and virtual meetings owing to COVID-19.^18^ Pandemic-induced change may potentially accelerate integration in other aspects of electronic/IT-based working, such as improved information and records sharing.^19^ The role of interface staff such as care navigators and social prescribers was emphasised by responders. For patients/service users and carers, these roles provide a single point of contact, aiding navigation of complex care systems and facilitating access to services beyond primary care and social services. This can contribute to a more seamless care transition between services and in utilising the system as a whole.^11^^,^^20^ The barriers to integration related to tensions are well documented, and include the substantial challenges of sharing records between organisations, poor communication (especially concerning older patients with multimorbidity transitioning between services), and the protectionism evident among organisations and professionals focused on their own specialist interests.^21^

### Implications for practice

This study highlights well-established drivers and barriers to integration that are also present in the primary care and social services context. If these are not adequately considered in the design and execution of new initiatives, progress towards integration is likely to continue at a slow pace. Failure to learn from previous models is concerning. It was found that it is essential to harness the potential of dynamic key individuals and/or teams to drive integration forward, and these findings add weight to the evidence base on the value of new interface roles. These models could potentially be expanded further, albeit with the important caveat that any wider rollout has greater efficacy if part of other systems-wide processes of integration. Efforts to progress integrated working have mainly concentrated on the clinical and professional aspects of integration located at the micro- and meso-levels of health and social care structures, indicating that integration has, to date, primarily been a bottom-up process. This suggests that there is potential scope to examine whether macro-scale integration can be increased at the higher organisational and strategic level across health and social care, and beyond. In doing so, this could contribute to a more holistic systems-wide approach to reform across England.
